# Extracellular Vesicle-functionalized Decalcified Bone Matrix Scaffolds with Enhanced Pro-angiogenic and Pro-bone Regeneration Activities

**DOI:** 10.1038/srep45622

**Published:** 2017-04-03

**Authors:** Hui Xie, Zhenxing Wang, Liming Zhang, Qian Lei, Aiqi Zhao, Hongxiang Wang, Qiubai Li, Yilin Cao, Wen Jie Zhang, Zhichao Chen

**Affiliations:** 1 Institute of Hematology, Union Hospital, Tongji Medical College, Huazhong University of Science and Technology, Wuhan 430022, P. R. China; 2 Department of Plastic and Reconstructive Surgery, Shanghai 9th People’s Hospital, Shanghai Jiao Tong University School of Medicine, Shanghai Key Laboratory of Tissue Engineering, National Tissue Engineering Center of China, Shanghai 200011, P. R. China; 3 Department of Hematology, the Central Hospital of Jingzhou, Jingzhou 434020, P. R. China; 4 Department of Hematology, the Central Hospital of Wuhan, Wuhan 430012, P. R. China

## Abstract

Vascularization is crucial for bone regeneration after the transplantation of tissue-engineered bone grafts in the clinical setting. Growing evidence suggests that mesenchymal stem cell (MSC)-derived extracellular vesicles (EVs) are potently pro-angiogenic both *in vitro* and *in vivo*. In the current study, we fabricated a novel EV-functionalized scaffold with enhanced pro-angiogenic and pro-bone regeneration activities by coating decalcified bone matrix (DBM) with MSC-derived EVs. EVs were harvested from rat bone marrow-derived MSCs and the pro-angiogenic potential of EVs was investigated *in vitro*. DBM scaffolds were then coated with EVs, and the modification was verified by scanning electron microscopy and confocal microscopy. Next, the pro-angiogenic and pro-bone regeneration activities of EV-modified scaffolds were evaluated in a subcutaneous bone formation model in nude mice. Micro-computed tomography scanning analysis showed that EV-modified scaffolds with seeded cells enhanced bone formation. Enhanced bone formation was confirmed by histological analysis. Immunohistochemical staining for CD31 proved that EV-modified scaffolds promoted vascularization in the grafts, thereby enhancing bone regeneration. This novel scaffold modification method provides a promising way to promote vascularization, which is essential for bone tissue engineering.

The repair and functional regeneration of extensive bone defects caused by trauma, tumor resections, or congenital deformities remain a challenge for modern medicine. Autologous and allogeneic bone grafting are commonly used to treat extensive bone defects, but these approaches suffer many problems such as donor site morbidity and limited availability^1^. Bone tissue engineering has the potential to overcome these drawbacks by culturing osteogenic cells in appropriate scaffold materials that provide an optimal environment for bone regeneration^2^. This approach has proved effective in animal models and in clinical cases^3^. However, despite significant progress, a number of problems remain unsolved, among which vascularization is one of the foremost challenges^4^. For extensive bone defects that initially lack vascularization, if adequate vascular supply cannot be established in time, the implanted cells within graft interior would suffer from hypoxia and apoptosis, resulting in a necrotic core in the graft^5^. To solve this problem, numerous strategies have been developed, including the delivery of angiogenesis-promoting growth factors, co-culture with endothelial cells, genetic modification of seeded cells, and surgical pre-vascularization techniques^6^,^7^,^8^,^9^. The enhanced bone regeneration observed in these studies showed that vascularization is essential for bone tissue engineering applications.

Extracellular vesicles (EVs), are membranous vesicles released from various cell types, and are now recognized as indispensable mediators of cell-to-cell communication^10^,^11^. EVs include both exosomes derived from the endosomal compartment and microvesicles (also called microparticles or ectosomes) derived directly from budding of the cell plasma membrane^12^. Being enclosed by a lipid bilayer, these vesicles contain both membrane components (including antigens, receptors, and lipid rafts) and cytoplasmic contents (including proteins, lipids, and nucleic acids) derived from their parent cells. EVs can thus be considered as miniature versions of a cell^10^. Unlike classic mechanisms of information exchange between cells via secreted soluble factors and cell-to-cell adhesion contact, EVs deliver their bioactive cargoes to the target cells. These bioactive cargoes can mediate genetic alteration of the target cells and lead to cell fate change^13^,^14^.

Recently, increasing attention has been focused on mesenchymal stem cell (MSC)-derived EVs. MSC-derived EVs are known for their potent pro-angiogenic effects^15^,^16^,^17^,^18^. Zhang *et al*. demonstrated that injection of EVs released from human umbilical cord-derived MSCs could significantly improve blood flow recovery in a rat model of hind-limb ischemia^16^. Bian *et al*. discovered that intra-myocardial injection of EVs released from human bone marrow-derived MSCs markedly enhanced blood flow recovery and restored cardiac systolic and diastolic function in a rat model of acute myocardial infarction^17^. In addition, Lopatina *et al*. reported that EVs released from human adipose tissue-derived MSCs could stimulate the angiogenic activity of human microvascular endothelial cells both *in vitro* and *in vivo*^18^.

Based on the reported pro-angiogenic effects of MSC-derived EVs, we speculated that scaffolds modified with MSC-derived EVs may enhance bone regeneration by promoting vascularization. In the present study, we investigated the pro-angiogenic effect of EVs secreted by rat bone marrow-derived MSCs *in vitro.* We also developed a novel EV-functionalized scaffold by coating decalcified bone matrix (DBM) with MSC-derived EVs. The pro-angiogenic and pro-bone formation effects of EV-modified scaffolds were investigated in a subcutaneous bone formation model using nude mice. The procedures for fabricating EV-functionalized DBM scaffolds and the experiment grouping design are shown in Fig. 1.

## Results

### Characterization of MSCs

Rat bone marrow-derived MSCs grew as plastic-adherent cells with a spindle shape at passage 3 (Fig. S1A). Under permissive culture conditions, MSCs underwent osteogenic, adipogenic, and chondrogenic differentiation, as demonstrated by alizarin red, oil red O, and safranin O staining, respectively (Fig. S1B–D), confirming their multi-lineage differentiation ability. Flow cytometric analysis was conducted to identify the expression of cell surface markers in cultured MSCs. As shown in Fig. S1E, MSCs were positive for mesenchymal markers (CD73 and CD105) and cell adhesion molecules (CD29, CD44 and CD90) and negative for hematopoietic markers (CD34 and CD45).

### Characterization of MSC-derived EVs

The morphology of EVs was observed using a scanning electron microscope (SEM). EVs were spheroidal, and their sizes were heterogeneous, with diameters in the range 100–1000 nm (Fig. 2A). These findings were consistent with those of other reports^19^. After being stained with the fluorescent dye carboxyfluorescein succinimidyl amino ester, EVs could be observed under a confocal microscope (Fig. 2B). For flow cytometric analysis, particles were defined as intact EVs if they were positively stained for calcein AM. As shown in Fig. 2C, the percentage of calcein-AM-positive freshly-isolated EVs was about 80%. We also investigated whether freeze/thaw cycles would damage EV integrity. Our data showed that one freeze-thaw cycle of EVs resulted in a minor reduction in calcein-AM staining, while multiple freeze-thaw cycles resulted in a dramatic reduction in calcein-AM staining (Fig. S2). We then investigated EV phenotype using flow cytometry. Figure 2D,E showed that EVs were positive for CD73, CD105, CD29, CD44, and CD90 expression and negative for CD34 and CD45 expression.

### EVs Promoted Proliferation, Migration, and Tube Formation of Human Umbilical Vein Endothelial Cells *in vitro*

To investigate the effect of EVs on the proliferation of human umbilical vein endothelial cells (HUVECs) *in vitro*, HUVECs were cultured in the presence of EVs. CCK-8 assays were performed after 1, 2, and 3 days of EV treatment. Compared to untreated cells, HUVECs grew faster in a dose-dependent manner in the presence of EVs at concentrations of 1, 20, and 50 μg/mL (Fig. 3A). These findings indicated that EVs promote the proliferation of HUVECs.

To determine the effect of EVs on the migration of HUVECs, scratch wound healing assays were performed. As shown in Fig. 3B, the scratched area was initially devoid of cells. After 12 h of incubation in the presence of 20 μg/mL EVs, HUVECs had already moved toward the cell-free area and had closed the scratch wound by about 52%. This response was much faster than that of cells in the untreated control wells. After 24 h of incubation, closure of the scratch with fully confluent HUVECs was observed in EV treated wells (Fig. 3B,C). In contrast, in the untreated control wells, HUVEC migration into the scratch area had closed the wound by only about 47%. These findings showed that EVs promote the migration of HUVECs.

Tube formation assays were conducted in Matrigel to determine whether EVs possess pro-angiogenic potential. Microscopic observation established that HUVECs formed tube-like structures after 12 h of incubation with 20 μg/mL EVs. In contrast, very few tube-like structures were observed in the control group (Fig. 3D,E).

### The Effect of EVs on the Proliferation, Apoptosis and Osteogenesis of MSCs

To investigate the effect of EVs on the proliferation of MSCs *in vitro*, MSCs were cultured in the presence of EVs. CCK-8 assays were performed after 1, 3, 5, 7 and 9 days of EV treatment. As shown in Fig. 4A, the growth curves of MSCs treated with EVs were similar to that of untreated MSCs. These findings indicated that EVs do not promote proliferation of MSCs.

Since a number of studies have demonstrated that MSC-derived EVs have anti-apoptotic effects on injured cells^20^,^21^, and seed cells in scaffolds often suffer from hypoxia and poor nutrient supply immediately after *in vivo* transplantation^22^,^23^, terminal transferase dUTP nick-end labeling (TUNEL) assays were performed to investigate whether EVs have anti-apoptotic effects on MSCs treated with hypoxia and serum deprivation. MSCs were cultured under the following three conditions: (1) with DMEM supplemented with 10% FBS in normoxia (control group); (2) with serum-free DMEM in hypoxia (Hy + SD group); (3) with serum-free DMEM supplemented with EVs in hypoxia (Hy + SD + EV group). As shown in Fig. 4B,C, in the control group, few TUNEL-positive cells were detected. In the Hy + SD group and Hy + SD + EV group, TUNEL-positive cells increased compared with the control group. Additionally, there was no significant difference in TUNEL-positive cells between the Hy + SD group and the Hy + SD + EV group (Fig. 4B,C). These data suggested that EVs have no major effect on the apoptosis of MSCs.

To determine whether EVs can promote osteogenic differentiation of MSCs, real-time quantitative polymerase chain reaction (qRT-PCR) analysis was conducted. For 10 days before qRT-PCR analysis, MSCs were incubated with growth media supplemented with EVs (EV group), with osteogenic inductive media (OS group), and with osteogenic inductive media supplemented with EVs (OS + EV group). MSCs cultured in growth media served as the control. The results showed that expressions of Runt-related transcription factor 2 (RUNX2), osteocalcin (OCN), and osteopontin (OPN) significantly increased in the OS group and in the OS + EV group compared with those in the control group. In addition, there were no significant differences in the expressions of RUNX2, OCN, and OPN between the EV group and the control group. These data suggest that EVs do not enhance osteogenic differentiation of MSCs (Fig. 4D).

### Characterization of EV-Modified Scaffolds

After coating DBM with carboxyfluorescein succinimidyl amino ester-labeled EVs, the distribution of EVs in the scaffold was inspected by a laser scanning confocal microscope. As shown in Fig. 5A, no fluorescent signals were observed in unmodified DBM scaffolds, whereas EVs with green fluorescence were distributed evenly in the modified scaffolds. Modification of the scaffold with EVs was further confirmed by SEM analysis (Fig. 5B). No EVs were observed on the unmodified scaffold, whereas EVs ranging from 100 to 1000 nm in diameter with intact membranes were observed on the microporous surface of the DBM scaffolds.

### EV-Modified Scaffolds Promote Bone Regeneration *in vivo*

To determine whether EV-modified scaffolds enhance bone regeneration *in vivo*, constructs of EV-modified or unmodified scaffolds seeded with or not seeded with MSCs were implanted subcutaneously into nude mice. To evaluate bone formation after 1 and 2 months of implantation, samples from all groups were harvested and scanned by micro-computed tomography (micro-CT). As shown in Fig. 6A,B, no significant differences were observed between the four groups in terms of macroscopic appearance and wet weight. After 1 month of implantation, bone volume (BV) and the bone volume to tissue volume ratio (BV/TV) in the MSCs + EV-modified scaffold (C + ES) group were increased compared with those in the other three groups (Fig. 6C,E). This relative difference between the C + ES group and the other groups was even stronger after 2 months, at which time the BV and BV/TV in the other three groups were significantly lower than those of the C + ES group (Fig. 6C,E). Thus, the C + ES group exhibited greater new bone formation compared with the control groups after 1 and 2 months of implantation. Interestingly, on observing the cross-sectional views near the center of the constructs (Fig. 6D), we found that the gray level (which indicates the degree of bone regeneration) was higher in the C + ES group than in the other groups. This result indicated that EV-modified DBM scaffolds enhanced bone regeneration in the central region of the grafts in particular.

### Histological and Immunohistochemical Analyses

After 2 months of implantation, the decalcified hematoxylin and eosin (HE)-stained specimens of the C + ES group revealed significantly more new bone formation, compared with the other groups (Fig. 7). In the C + ES group, osteoblast-like cells were retracted and separated from each other and from their underlying matrix. These cells lined the surface of bone trabecular of DBM scaffold, producing bone matrix. Fewer osteoblast-like cells were found in the MSCs + unmodified scaffold (C + S) group, and very few were found in the EV-modified scaffold (ES) group and the unmodified scaffold (S) group (Fig. 7), which indicated reduced levels of new bone formation.

To investigate whether EV-modified DBM scaffolds promoted vascularization *in vivo*, tissue slices from each group were stained with anti-CD31 (which is a marker for vascular endothelial cells) antibody by immunohistochemical staining. Specimens from all four groups showed positive labeling for CD31. An observably enhanced angiogenic response was evident in the C + ES group, leading to a significantly higher average number of CD31-positive vessels than those seen in the other groups. Importantly, the ES group had twice the average number of CD31-positive vessels compared with the S group (Fig. 8A,B). These results confirmed the ability of EVs to promote vascularization *in vivo*.

## Discussion

Bone tissue engineering provides a promising approach to the treatment of extensive bone defects. However, because of limitations in nutrient and oxygen diffusion caused by poor vascularization, necrosis often occurs in the cores of implanted tissue-engineered bone grafts. Such necrosis greatly hinders the clinical application of this strategy^4^. Consequently, it is clear that vascularization plays a vital role in the healing and functional regeneration of damaged or diseased bone tissues. Observations made in studies of several ischemic disorders indicate that MSC-derived EVs possess potent pro-angiogenic activities^16^,^17^. Therefore, we coated DBM scaffolds with MSC-derived EVs and showed that the EV-modified scaffolds promoted bone regeneration by accelerating vascularization.

It is now recognized that EVs are shed by various cell types in both resting and activated states^24^. In the current study, what we isolated from the culture media of MSCs were mainly microvesicles (also called microparticles or ectosomes) that were released by direct budding of the cell plasma membrane. Exosomes which are released by the fusion of multivesicular bodies with plasma membrane could not be pelleted under our centrifugation conditions (20,000 × g)^25^. We confirmed that rat bone marrow-derived MSCs released EVs with diameters in the range 100–1000 nm and with the same immunophenotype as their parent cells. To avoid contamination with EVs derived from fetal calf serum, the cell culture media was replaced by serum-free media 24 h before EVs were harvested. We found that MSC-derived EVs stimulated cell proliferation, cell migration, and tube formation of HUVECs *in vitro*, which is consistent with the findings of previous reports^16^,^17^,^18^. The pro-angiogenic activity of MSC-derived EVs has therefore been confirmed. Interestingly, we found that the number of EVs released during serum-free stimulation was nearly twice the number of EVs released in the presence of serum (data not shown). This is also in accordance with the findings of other reports^16^,^17^.

An elementary requirement for modifying DBM scaffolds with EVs is to coat the scaffolds tightly with EVs so that they cannot be easily dislodged. Bruno *et al*. reported that EVs from human bone marrow-derived MSCs showed expression of several adhesion molecules, including Integrin α-5^26^. In addition, Narayanan *et al*. discovered that MSC-derived EVs could bind to matrix proteins such as type I collagen and fibronectin^27^. Consequently, we coated DBM scaffolds with fibronectin before EV coating to promote the adherence of EVs to the scaffolds. Confocal microscopy showed that EVs were evenly distributed throughout the scaffold even after several washes with phosphate buffer saline (PBS). Additionally, SEM analysis revealed that EVs remained intact for extended time intervals when adhered to scaffolds, which is important because membrane integrity is crucial for EV-mediated cell-to-cell communication^28^.

EV-modified scaffolds had significant effects on bone regeneration *in vivo*. From micro-CT and histological analyses, the C + ES group revealed significantly more new bone formation compared with the other groups. While in the ES group which had no implanted seed cells, no significant bone formation-promoting effect was observed. Previous studies showed that the osteogenic cells that formed new bone in subcutaneous bone formation model were mainly of donor origin rather than originating from the local microenvironment^29^,^30^. Thus, even though more CD31-positive vessels were formed in the ES group, no significant bone formation was observed in this group. *In vitro* experiments showed that MSC-derived EVs had no major effect on the proliferation, apoptosis and osteogenesis of MSCs, indicating that EV-modified scaffolds promote bone regeneration mainly by accelerating vascularization. Whether MSC-derived EVs have anti-apoptotic effects *in vivo* is worth to be investigated in future. In a preliminary study, we have investigated the scaffolds modified by rat dermal fibroblasts-derived EVs, no significant improvement of bone formation was observed by modification with fibroblast derived-EVs (unpublished data). These findings suggest that the beneficial effects on bone formation found in our experiments were specific to MSC-derived EVs. A comparison of the bioactive contents (e.g., growth factors, mRNAs, and miRNAs) of MSC-derived EVs and fibroblast-derived EVs warrants further investigation.

One approach commonly taken by researchers to promote vascularization in bone tissue engineering is to achieve sustained release of growth factors from the scaffolds. A wide variety of synthetic microparticles loaded with angiogenic or multiple growth factors have been used to accomplish this goal^22^. For example, Patel *et al*. incorporated gelatin microparticles loaded with vascular endothelial growth factor and bone morphogenetic protein-2 into poly (propylene fumarate) scaffolds and found that this modification resulted in increased bone regeneration^31^. EVs have several inherent advantages compared with synthetic microparticles. First, they are naturally occurring vesicles released from their parent cells. Growing evidence suggests that allogeneic, or even xenogeneic EVs, are well tolerated in immune-competent animals^32^,^33^. Therefore, the use of allogeneic or even xenogeneic EVs might help to solve the problem of a severe shortage of appropriate donor cells. Second, our study showed that MSC-derived EVs possess potent pro-angiogenic activity both *in vitro* and *in vivo*. Others have reported that EVs secreted by MSCs derived from other tissue sources such as umbilical cord and adipose tissue are enriched in angiogenesis-related growth factors, mRNAs, and miRNAs^15^,^34^.The pro-angiogenic components of EVs secreted by rat bone marrow derived-MSCs are under investigation in our laboratory. By delivering these bioactive cargoes to their recipient cells, EVs might mediate genetic alteration of the recipient cells and exert pro-angiogenic effects^32^. Third, increasing interest has recently focused on genetic manipulation of the content of EVs^35^. In that way, it may be possible to enrich EVs with mRNAs, miRMAs or biomolecules that favor tissue regeneration.

Although the advantages of EVs listed above inspire great optimism for the potential of EVs in scaffold fabrication, many challenges remain before widespread application of EVs. For example, large-scale production methods for EVs must be developed. It has been reported that cells under stimulation, such as those in serum-free culture or experiencing hypoxia, release larger numbers of EVs^16^,^17^. However, such stimulation would unavoidably induce a stress response, probably leading to secretion of EVs with altered bioactive cargoes. Consequently, it will be necessary to compare the EVs released from different cells under different culture conditions^25^. Additionally, developing effective storage methods for EVs and EV-modified scaffolds would be helpful to facilitate widespread clinical application. Evidence suggests that EVs are relatively resistant to freeze/thaw cycles and even hypotonic environments^25^. However, our data and other evidence suggest that repeated freeze/thaw cycles would damage EV integrity^36^. The long-term preservation of EVs and EV-modified scaffolds is under investigation in our laboratory. Finally, despite many studies having suggested that EVs can be safely administered in animals, biosafety is the most important issue for new therapeutic approaches.

To our knowledge, this is the first report of EVs used for scaffold modification. Although many issues remain to be addressed, this novel scaffold modification method provides a promising way to promote vascularization, which is crucial for bone tissue engineering. This method may also be applicable in the regeneration of other tissues or organs.

## Materials and Methods

### Cell Culture

#### Primary Culture of Bone Marrow-derived MSCs

The study protocol was approved by the Ethics Committee of Huazhong University of Science and Technology. All experiments involving animals were performed in accordance with relevant guidelines and regulations. Ten-day-old male Sprague-Dawley rats were killed by cervical dislocation, and bone marrow-derived MSCs were isolated as previously described with some modifications^37^. Briefly, under aseptic conditions, femora and tibia were removed and bone marrow was flushed out using a 1-mL syringe. A suspension of single bone marrow cells was obtained by repeated aspiration. Cells were seeded into culture dishes and maintained in Dulbecco’s modified Eagle’s media (DMEM; Hyclone, Waltham, MA, USA) supplemented with 10% (v/v) fetal bovine serum (FBS; Hyclone) and 1% (v/v) penicillin–streptomycin antibiotic (Gibco, Grand Island, NY, USA) in a humidified incubator under an atmosphere of 5% CO_2_/95% air at 37 °C. After 48 h, the culture media was replaced and non-adherent cells were removed. Cell passaging was performed until the monolayer of adherent cells reached 70–80% confluence.

### Culture of HUVECs

HUVECs were purchased from the American Type Culture Collection (ATCC, Rockville, MD, USA). The cells were cultured in DMEM supplemented with 10% FBS and 1% penicillin–streptomycin antibiotic in a humidified incubator under an atmosphere of 5% CO_2_/95% air at 37 °C. Cell passaging was performed when the monolayer of adherent cells reached 70–80% confluence.

### Characterization of bone marrow-derived MSCs

#### Immunophenotype

Bone marrow-derived MSCs from the third passage were harvested for flow cytometric analysis. Aliquots of 4 × 10^5^ cells were suspended in 200 μL washing buffer (PBS containing 2% FBS). Cells were then incubated on ice with phycoerythrin (PE)- or peridinin chlorophyll protein (PerCP)-conjugated anti-CD73, CD105, CD29, CD44, CD90, CD34, and CD45 antibodies (BD Bioscience, San Jose, CA, USA). PE- and PerCP-conjugated isotype-matched immunoglobulins were used as controls. After 30 min of incubation, cells were washed three times with PBS and then analyzed on a flow cytometer (BD Bioscience). Flow cytometric data were analyzed with FlowJo software version 7.6 (TreeStar Inc., Ashland, OR, USA).

### Multilineage Differentiation of MSCs

The osteogenic, adipogenic, and chondrogenic differentiation potential of MSCs was measured as previously described with some modifications^38^. All chemicals were purchased from Sigma (St Louis, MO, USA) unless otherwise stated. For osteogenic differentiation, MSCs were cultured in growth media supplemented with 10 mM β-glycerophosphate, 0.1 μM dexamethasone, and 50 μM ascorbic acid. The media was changed twice a week for 2 weeks. After induction, cells were fixed with 10% formalin for 20 min and stained with alizarin red for 20 min at 37 °C.

For adipogenic differentiation, MSCs were cultured in growth media supplemented with 5 μg/mL insulin, 200 μM indomethacin, 1 μM dexamethasone, and 0.5 mM 3-isobutyl-1-methylxanthine. The media was changed twice a week for 3 weeks. After induction, cells were fixed with 10% formalin for 20 min and stained with 0.5% oil red O in methanol for 20 min.

For chondrogenic differentiation, MSCs were pelleted and cultured in growth media supplemented with 0.1 μM dexamethasone, 0.17 mM ascorbic acid, 1 mM sodium pyruvate, 0.35 mM l-proline, 1% insulin-transferrin sodium-selenite, 1.25 mg/mL bovine serum albumin, 5.33 μg/mL linoleic acid, and 0.01 μg/mL transforming growth factor-β (Cell Science, Canton, MA, USA). The media was changed twice a week for 4 weeks. After incubation, the micromass pellets were fixed with 10% formalin, embedded in paraffin, and then sectioned in 10-μm slices. The samples were dewaxed, rehydrated, and stained with safranin O.

### Isolation and Characterization of MSC-derived EVs

MSC-derived EVs were harvested as previously described with some modifications^39^. Briefly, rat bone marrow-derived MSCs from the third and fourth passages were used for EV isolation. When cells reached 70–80% confluence, they were washed three times with PBS and then the cell culture media was replaced by serum-free DMEM. After an additional 24 h of incubation, the culture media was collected and centrifuged at 2000 × *g* for 20 min to remove cell debris. The viability of cells incubated without serum for 24 h was >95% as detected by trypan blue exclusion. Subsequently, the supernatant was centrifuged at 20,000 × *g* for 1 h at 4 °C. The supernatant was removed, and the pelleted EVs were washed with ice-cold PBS and pelleted again by centrifugation at 20,000 × *g* for 1 h at 4 °C. Finally, the supernatant was removed, and the pelleted EVs were resuspended in PBS and stored at −80 °C until they were used in experiments. In the present study, we avoided repeated freezing and thawing EVs, EVs used for *in vitro* and *in vivo* experiments were obtained upon only one freeze/thaw cycle. The total protein content of EVs was quantified using a BCA protein assay kit (Beyotime, Beijing, China) following the manufacturer’s instructions.

To confirm the presence of EVs, random samples of EVs isolated by differential centrifugation were visualized by SEM and confocal laser scanning microscopy. SEM analysis of EVs was performed as previously described with some modifications^40^. Briefly, the EVs were fixed with 2.5% glutaraldehyde in PBS for 2 h. The fixed EVs were washed three times with PBS and then dehydrated with an ascending sequence of ethanol. After evaporation of the ethanol, the samples were dried at room temperature on a glass substrate, followed by gold-palladium sputtering. The samples were then observed by SEM (Hitachi, Tokyo, Japan). For confocal microscopic analysis, EVs were stained with carboxyfluorescein succinimidyl amino ester (Beyotime) following the manufacturer’s instructions, and then observed with a confocal microscope (Leica, Wetzlar, Germany).

To investigate whether repeated freeze/thaw cycles would damage EV integrity, EVs were subjected to one or three rounds of freeze/thaw cycles using an ultra low temperature freezer (Thermo Scientific, Waltham, MA, USA) and a 37 °C water bath. After the final thaw, EVs were stained with calcein-AM (Molecular Probes, Eugene, OR, USA) as previously described^36^. Calcein AM was used to avoid staining of cell debris, because only intact EVs would fluoresce. Then EVs were analyzed on a flow cytometer (BD Bioscience), and freshly-isolated EVs were used as control. To determine the phenotypic profile of EVs, EVs were co-stained with calcein AM (Molecular Probes) and PE- or PerCP-conjugated anti-CD73, CD105, CD29, CD44, CD90, CD34, and CD45 antibodies (BD Bioscience) and analyzed on a flow cytometer (BD Bioscience). PE- and PerCP-conjugated isotype-matched immunoglobulins were used as controls. Flow cytometric data were analyzed using FlowJo software version 7.6 (TreeStar Inc.).

### Cell Proliferation Assay

HUVECs were seeded at 1000 cells/well in a 96-well plate and cultured in 10% FBS-supplemented DMEM in the presence of graded doses of MSC-derived EVs or PBS (control). At 1, 2, and 3 days post-treatment, the cell culture media was removed and 10 μL of cell counting kit-8 (CCK-8, Beyotime) solution and 100 μL of fresh cell culture media were added. The culture was maintained for another 2 h and the optical density (OD) at 450 nm was measured by a microplate reader (Thermo Scientific).

MSCs were seeded at 800 cells/well in a 96-well plate and cultured in 10% FBS-supplemented DMEM in the presence of graded doses of MSC-derived EVs or PBS (control). At 1, 3, 5, 7 and 9 days post-treatment, CCK-8 assays were performed as mentioned above.

### Scratch Wound Healing Assay

HUVECs were seeded at 2 × 10^5^/well in a six-well plate and cultured in 10% FBS-supplemented DMEM. After the monolayer of adherent cells reached 100% confluence, a wound was created by manually scraping the cell monolayer with a P200 pipette tip. Then the HUVECs were cultured in DMEM supplemented with 0.5% bovine serum albumin (BSA) for another 12 and 24 h with the addition of EVs (20 μg/mL) or PBS (control). Images were captured by a phase-contrast microscope (Olympus, Tokyo, Japan) at 0, 12, and 24 h after the scratch wound was made. The area of the wounded cell-free region was recorded for evaluation. Five independent experiments were performed.

### Tube Formation Assay

HUVECs (5 × 10^4^ per well) were seeded into Matrigel (BD Biosciences)-coated wells in a 24-well plate and cultured in DMEM supplemented with 0.5% BSA in the presence of EVs or PBS (control). Three replicated wells were set up for each group. After incubation for 12 h, tube formation was examined with a phase-contrast microscope (Olympus) and the total length of the network was evaluated in five randomly selected fields for each well. The total length of the network was measured using Image-Pro Plus 6.0 software (Media Cybernetics, Silver Spring, MD, USA), and expressed as a ratio to that of the respective control.

### TUNEL assay

To determine whether EVs have anti-apoptotic effects on MSCs treated with hypoxia and serum deprivation, MSCs were cultured under the following three conditions: (1) with DMEM supplemented with 10% FBS in normoxia (control group); (2) with serum-free DMEM in hypoxia (Hy + SD group); (3) with serum-free DMEM supplemented with 20 μg/mL EVs in hypoxia (Hy + SD + EV group). For normoxic condition, the cells were cultured in an atmosphere containing 5% CO_2_, while for hypoxia, the cells were maintained in a gas mixture composed of 94% N_2_, 5% CO_2_, and 1% O_2_. Three replicated slides were set up for each conditions. The cultures were maintained for 24 h. Apoptosis was analyzed by TUNEL staining, using a kit (Roche, Basel, Switzerland) as per the manufacturer’s instructions. In brief, after 24 h of incubation, the cells were fixed by 4% formaldehyde/PBS at room temperature for 20 min and subjected to TUNEL assay according to the manufacturer’s protocol. The slides were then counterstained with 4′,6-diamidino-2-phenylindole (DAPI, Beyotime). TUNEL-positive cells was examined with a phase-contrast microscope (Olympus) and the total number of TUNEL-positive cells was evaluated in five randomly selected fields for each slide.

### qRT-PCR

To determine the effect of EVs on the expression of osteogenesis-related genes in MSCs, qRT-PCR analysis was conducted. MSCs were cultured in growth media supplemented with EVs (EV group), osteogenic inductive media (OS group), and osteogenic inductive media supplemented with EVs (OS + EV group) for 10 days before RNA extraction. MSCs cultured in growth media served as the control. To eliminate the effect of EVs that had not entered the target cells, the target cells were washed three times with PBS. Total RNA was extracted from MSCs with Trizol (Invitrogen, Carlsbad, CA, USA). Complementary DNA (cDNA) was synthesized from 1 μg total RNA per sample using a reverse transcription kit (Takara, Tokyo, Japan). The cDNA was amplified using a Power SYBR Green PCR master mix (Applied Biosystems, Foster City, CA, USA) in a real-time thermal cycler (Mx3000PTM QPCR System, Stratagene, La Jolla, CA, USA) and each measurement was repeated in triplicate. The comparative Ct method was used for relative measurement of gene expression level against the glyceraldehyde 3-phosphate dehydrogenase (GAPDH) gene. The primers used in qRT-PCR analysis are listed in Table S1.

### Fabrication and Characterization of EV-Modified DBM scaffolds

DBM scaffolds were prepared from bovine limbs as previously described^41^. The scaffolds were cut into 3-mm cubes. After being immersed in 75% ethanol for 2 h, the scaffolds were washed three times with PBS and then coated with 10 μg/mL fibronectin (Sigma) overnight at 37 °C. Each scaffold was then loaded with 20 μg of EVs suspended in 20 μL PBS and incubated for 4 h at 37 °C. The scaffolds were then air dried and stored at −80 °C until they were used in experiments.

To evaluate the overall distribution of EVs on the scaffolds, the scaffolds were coated with carboxyfluorescein succinimidyl amino ester-labeled EVs and stored at −80 °C as described above. Then the EV-modified scaffolds were thawed and washed three times with PBS and observed under a confocal laser scanning microscope. Unmodified scaffolds served as the control. For SEM analysis, EV-modified scaffolds were thawed after being stored at −80 °C, and then fixed with 2.5% glutaraldehyde in PBS for 2 h. Unmodified scaffolds served as the control. The fixed scaffolds were washed three times with PBS and then dehydrated with an ascending sequence of ethanol. After evaporation of the ethanol, the samples were dried at room temperature and then observed using a SEM after gold–palladium sputtering.

### Bone Regeneration *in vivo*

**Figure 1:**
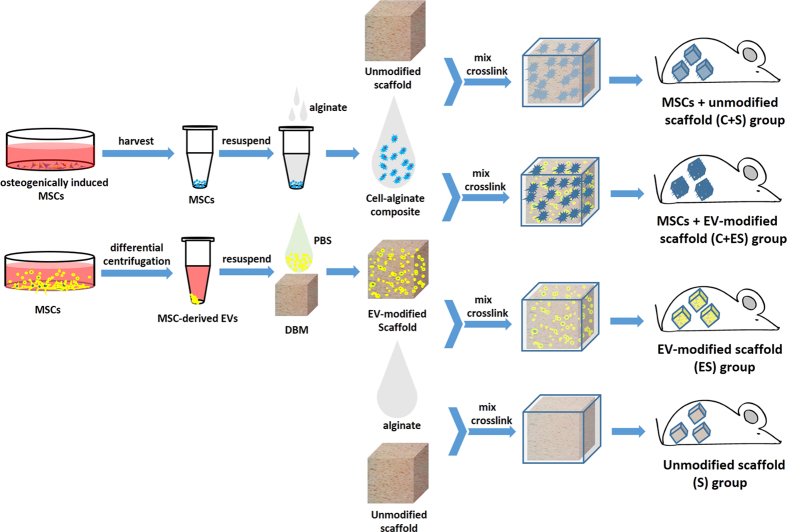
Schematic of *in vivo* bone regeneration procedures and the design of the four experimental groups.

**Figure 2:**
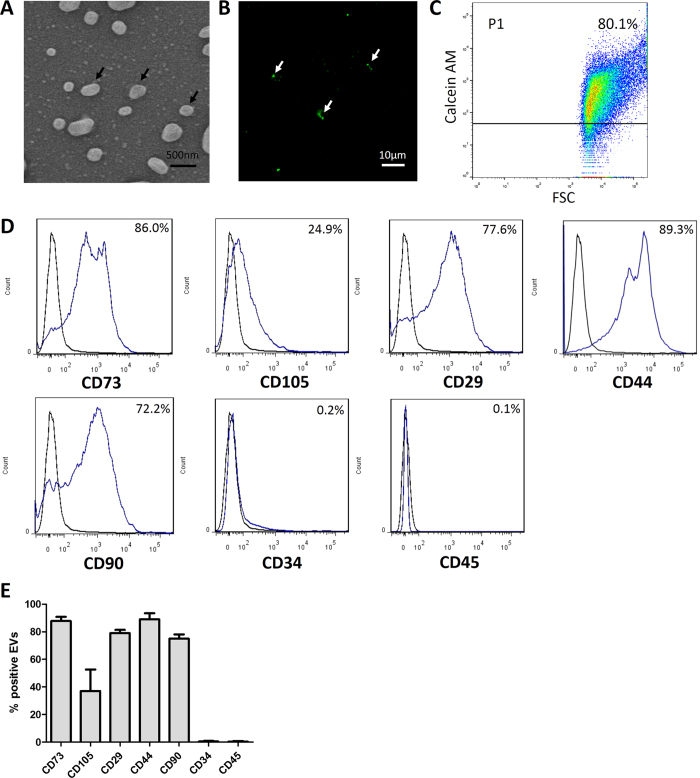
Characterization of MSC-derived EVs. (**A**) A representative SEM image of EVs (arrows) ranging in diameter from 100 to 1000 nm. (**B**) A representative confocal microscope image of carboxyfluorescein succinimidyl amino ester-labeled EVs with green fluorescence (arrows). (**C**) Representative dot plot showing EV size distribution and calcein AM positive rate. (**D**) Representative graphs of EV surface marker expression analyzed by flow cytometry. (**E**) Quantitative analysis of the flow cytometric data (n = 3).

**Figure 3:**
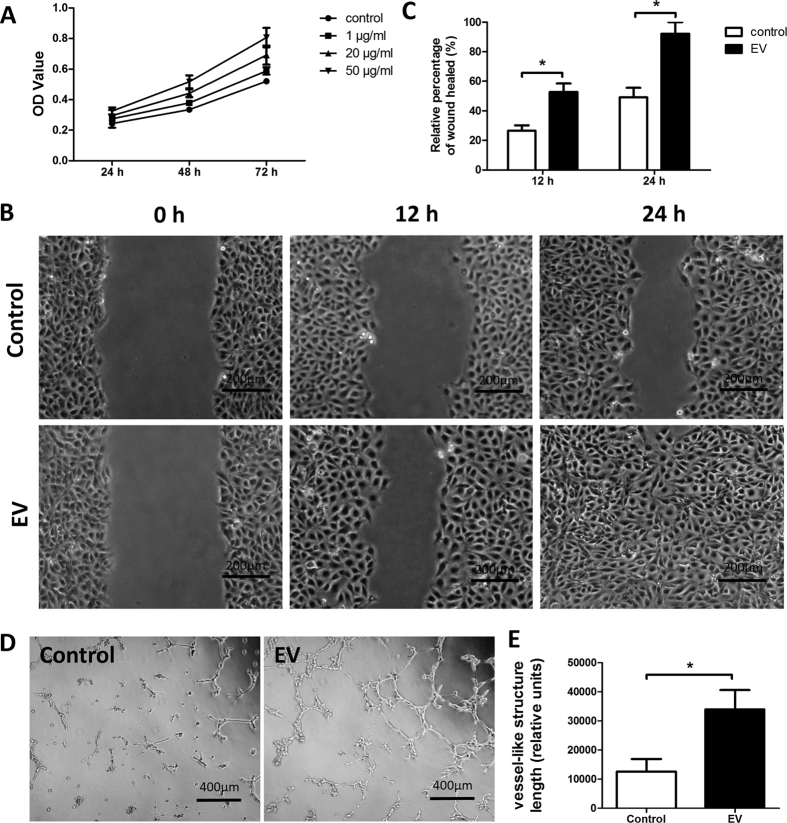
MSC-derived EVs promoted proliferation, migration, and tube formation of HUVECs. (**A**) The proliferation of HUVECs cultured in the presence of graded concentrations of EVs or PBS (control), n = 5. (**B**) Representative images of a scratch wound healing assay. (**C**) Quantitative analysis of the percentage of wound healing with or without EV stimulation, n = 5, *p < 0.05. (**D**) Representative images of a tube formation assay. (**E**) Quantitative analysis of the total length of tube-like structures with or without EV stimulation. Three replicated wells for each group and five randomly selected views from each well were analyzed, *p < 0.05.

**Figure 4:**
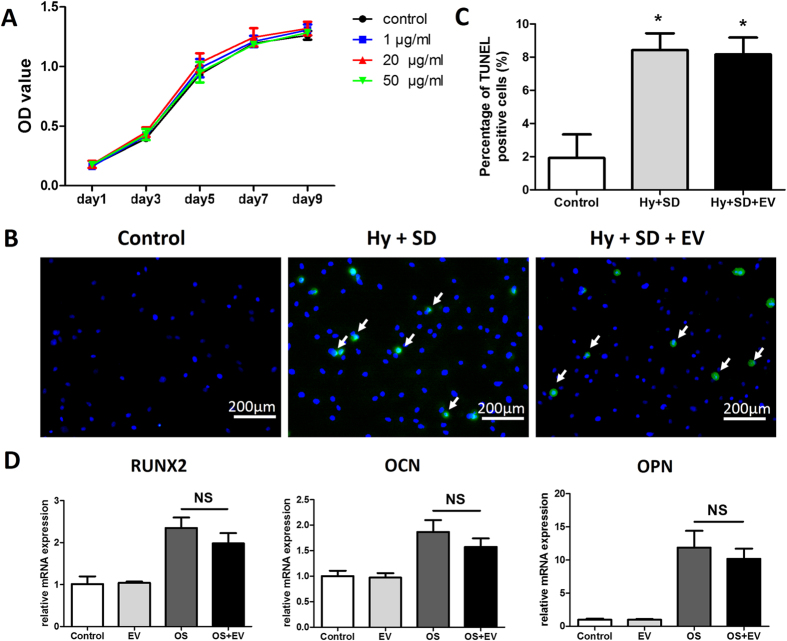
The effect of EVs on the proliferation, apoptosis and osteogenesis of MSCs. (**A**) The proliferation of MSCs cultured in the presence of graded concentrations of EVs or PBS (control), n = 5. (**B**) Representative images of TUNEL assay. (**C**) Quantitative analysis of the percentage of TUNEL-positive cells. Three replicated slides for each group and five randomly selected views from each slide were analyzed, *p < 0.05. (**D**) qRT-PCR analysis of gene expression for Runx2, OPN, and OCN. Experiments were carried out on three samples for each group. NS, not significant at the level of 0.05.

**Figure 5:**
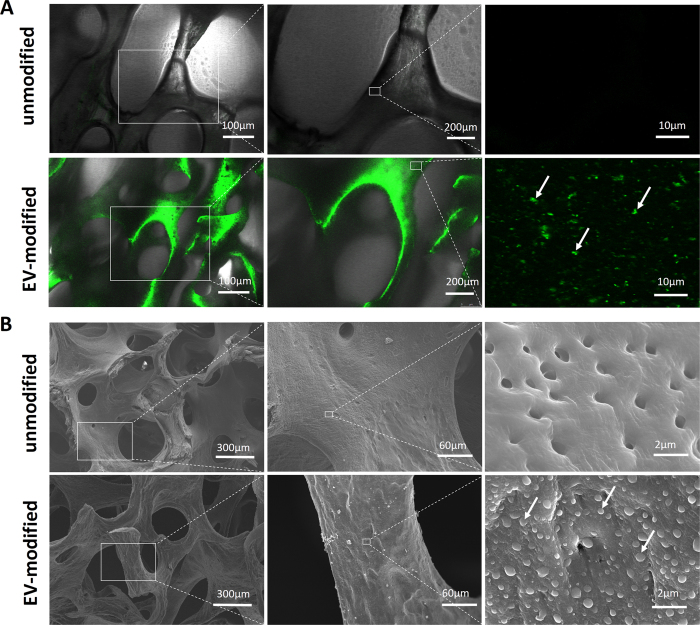
Characterization of EV-modified scaffolds. (**A**) Representative confocal microscopic images of unmodified and EV-modified DBM scaffolds at different magnifications. Arrows indicate carboxyfluorescein succinimidyl amino ester-labeled EVs. (**B**) Representative SEM images of unmodified and EV-modified DBM scaffolds at different magnifications. Arrows indicate EVs.

**Figure 6:**
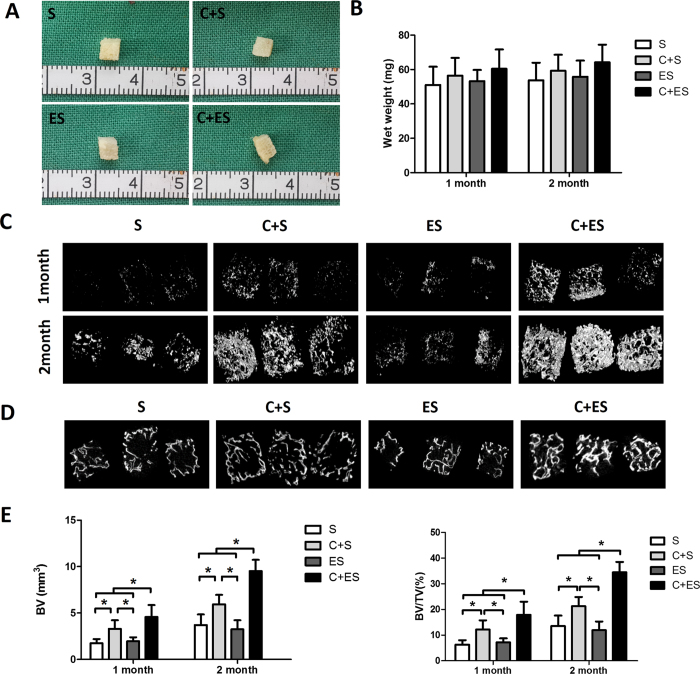
Micro-CT analysis of bone formation showed that EV-modified scaffolds could enhance bone regeneration *in vivo*. (**A**) Macroscopic views of the grafts after 2 months of implantation. (**B**) Quantitative analysis of wet weight of the grafts in each group (n = 5); no significant differences were observed between the groups. (**C**) Three-dimensional reconstruction of micro-CT images of the grafts in each group at 1 and 2 months. (**D**) Cross-sectional views of the central region of the grafts scanned by micro-CT at 2 months. (**E**) Statistical analysis of bone volume (BV) and bone volume to tissue volume ratio (BV/TV) in each group at 1 and 2 months (n = 5 per group per time point), *p < 0.05.

**Figure 7:**
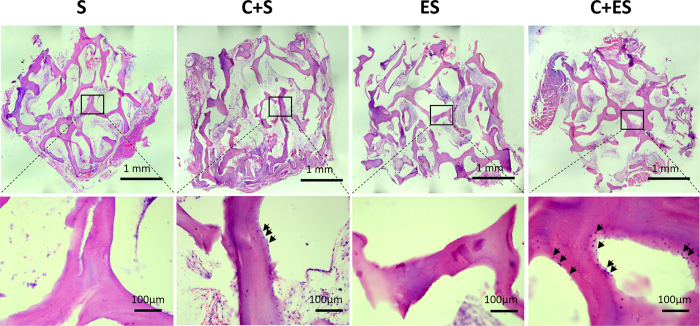
Histological analysis revealed that higher levels of new bone formation were observed in the C + ES group than in the other three groups. Representative images of HE staining of the grafts in each group after 2 months of implantation at low (upper panel) and high (lower panel) magnification. Arrows: osteoblast-like cells.

**Figure 8:**
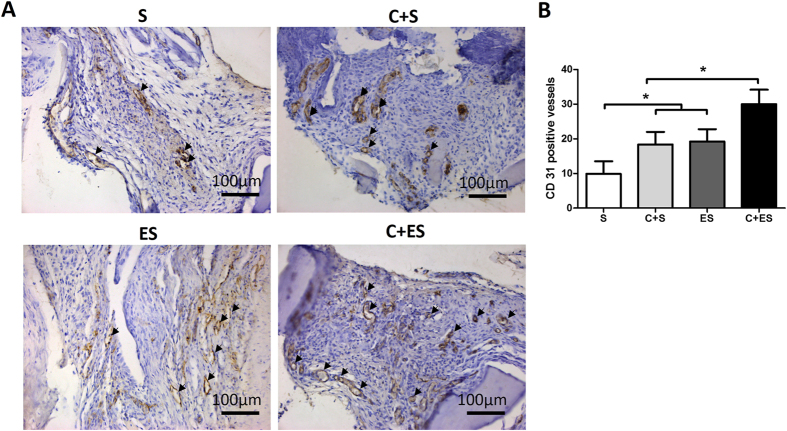
Immunohistochemical staining for CD31 showed an increase of vessel formation in the C + ES group compared with the other groups. (**A**) Representative views of CD31-positive vessels (arrows) in the grafts from each group. (**B**) Quantitative analysis of CD31-positive vessels in each group after 2 months of implantation. Three samples in each group and five views from each sample were analyzed, *p < 0.05.

To investigate the effects of the EV-modified scaffolds on bone regeneration *in vivo*, four groups were prepared and implanted subcutaneously into 4-week-old male nude mice (n = 10 for each group). As shown in Fig. 1, the four groups were: (1) The C + ES group. MSCs at passage three were osteogenically induced (as described above) for 10 days on culture plates. Cells were harvested and then mixed with 1.5% sodium alginate solution at a final cellular density of 2 × 10^7^/mL. Twenty microliters of cell–alginate composite solution was seeded onto each EV-modified scaffold. Then the cell–scaffold constructs were immersed in 100 mM CaCl_2_ solution for about 2 min to allow cross-linking. (2) The C + S group. Unmodified scaffolds were coated with 10 μg/mL fibronectin overnight at 37 °C. Then each scaffold was loaded with 20 μL of cell–alginate composite solution and cross-linked with CaCl_2_ solution. (3) The ES group. Each EV-modified scaffold was loaded with 20 μL of 1.5% sodium alginate solution and cross-linked with CaCl_2_ solution. (4) The S group. Unmodified scaffolds were coated with 10 μg/mL fibronectin overnight at 37 °C. Then each scaffold was loaded with 20 μL of 1.5% sodium alginate solution and cross-linked with CaCl_2_ solution.

After 1 and 2 months of implantation, animals were killed by an overdose of anesthesia. Specimens were harvested for micro-CT analysis, histological analysis and immunohistochemical analysis.

### Micro-CT Analysis

Samples were fixed in 4% paraformaldehyde and subjected to micro-CT scanning (μCT-80, Scanco Medical, Bassersdorf, Switzerland). The region of interest (ROI) was set as a cylinder (36 mm in diameter and 5 mm in height) including all the samples from a single group, and was three-dimensionally reconstructed. Quantitative morphometric analysis of the ROI was performed automatically by micro-CT auxiliary software (Volume Graphics GmbH, Heidelberg, Germany). The parameters obtained included BV and BV/TV.

### Histological and Immunohistochemical Analyses

After micro-CT analysis, specimens were decalcified with 10% ethylene diamine tetraacetic acid solution for 1 week, dehydrated through an ethanol series, cleared with xylene, and then embedded in paraffin. The specimens were cut into 10-μm-thick sections and stained with HE.

Expression of CD31 was detected using rabbit anti-mouse CD31 monoclonal antibody (Abcam, Cambridge, UK). This was followed by treatment with horseradish peroxidase-conjugated goat anti-rabbit antibody (Invitrogen) and color development with diaminobenzidine tetrahydrochloride (Santa Cruz, CA, USA). Five randomly selected fields from each tissue section (n = 3/group) were captured by a light microscope (Olympus). The number of CD31-positive blood vessels was calculated by Image-Pro Plus software (Media Cybernetics).

### Statistical Analysis

All data collected were presented as mean ± standard deviation. Data were analyzed using one-way analysis of variance or Student’s t-test, and p < 0.05 was considered statistically significant.

## Additional Information

**How to cite this article:** Xie, H. *et al*. Extracellular Vesicle-functionalized Decalcified Bone Matrix Scaffolds with Enhanced Pro-angiogenic and Pro-bone Regeneration Activities. *Sci. Rep.* **7**, 45622; doi: 10.1038/srep45622 (2017).

**Publisher's note:** Springer Nature remains neutral with regard to jurisdictional claims in published maps and institutional affiliations.

## Supplementary Material

Supplementary Data
