# Supplementary material for: Extracellular Vesicle-functionalized Decalcified Bone Matrix Scaffolds with Enhanced Pro-angiogenic and Pro-bone Regeneration Activities
Source: Sci Rep. 2017 Apr 3;7:45622. doi: 10.1038/srep45622 (PMC5377422; doi:10.1038/srep45622)
Supplement: Supplementary Data [file srep45622-s1.pdf]

Supplementary data

Extracellular Vesicle-functionalized Decalcified Bone Matrix Scaffolds with Enhanced Pro-angiogenic and Pro-bone Regeneration Activities

Hui Xie[a, 1], Zhenxing Wang[b, 1], Liming Zhang[c], Qian Lei[a], Aiqi Zhao[a], Hongxiang Wang[a], Qiubai Li[a], Yilin Cao[b], Wen Jie Zhang[b, *], Zhichao Chen[a, *]

[a] Institute of Hematology, Union Hospital, Tongji Medical College, Huazhong University of Science and Technology, Wuhan 430022, P. R. China

[b] Department of Plastic and Reconstructive Surgery, Shanghai 9th People's Hospital, Shanghai Jiao Tong University School of Medicine, Shanghai Key Laboratory of Tissue Engineering, National Tissue Engineering Center of China, Shanghai 200011, P. R. China

[c] Department of Hematology, the Central Hospital of Jingzhou, Jingzhou 434020, P. R. China

[d] Department of Hematology, the Central Hospital of Wuhan, Wuhan 430012, P. R. China

[1] These authors contributed equally to this work.

[*] Address correspondence to Zhichao Chen (chenzhichao@hust.edu.cn) and Wen Jie Zhang (wenjieboshi@aliyun.com).

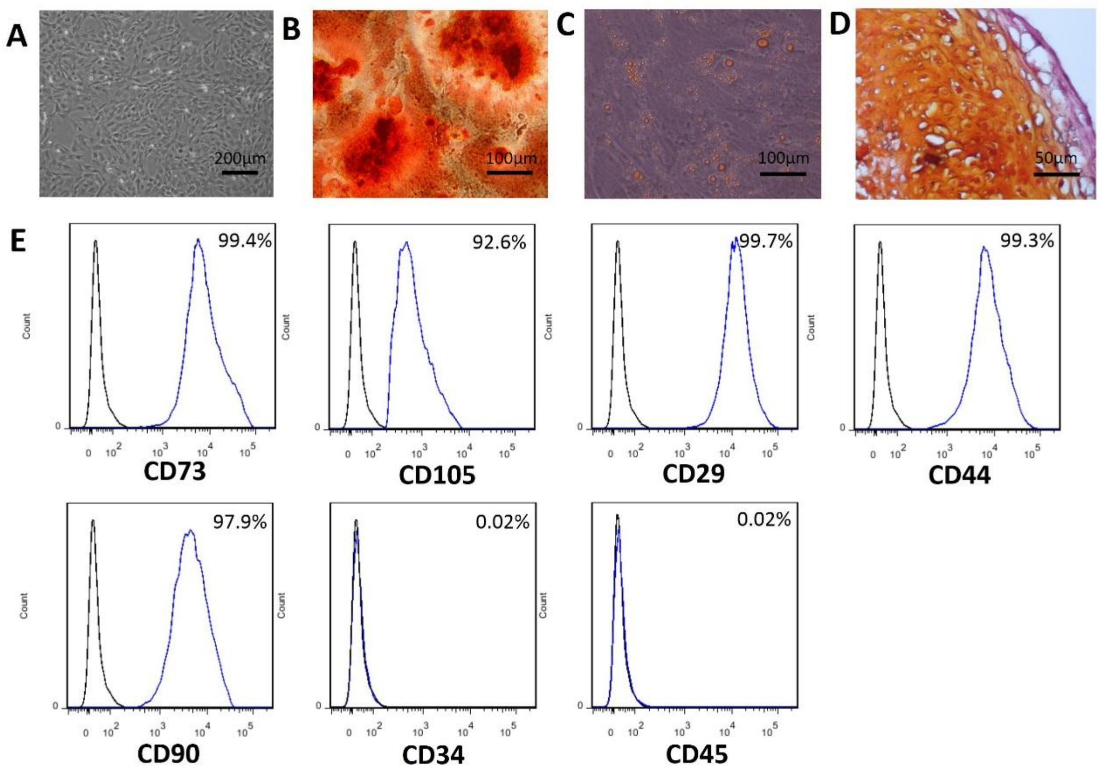

Fig. S1. Characterization of rat bone-marrow-derived MSCs. (A) Typical morphology of MSCs. (B) Alizarin red staining of MSCs after 2 weeks of osteogenic induction. (C) Oil red O staining of MSCs after 3 weeks of adipogenic induction. (D) Red safranin O staining of the MSC-derived micromass after 4 weeks of chondrogenic induction. (E) Representative graphs of cell surface marker expression analyzed by flow cytometry.

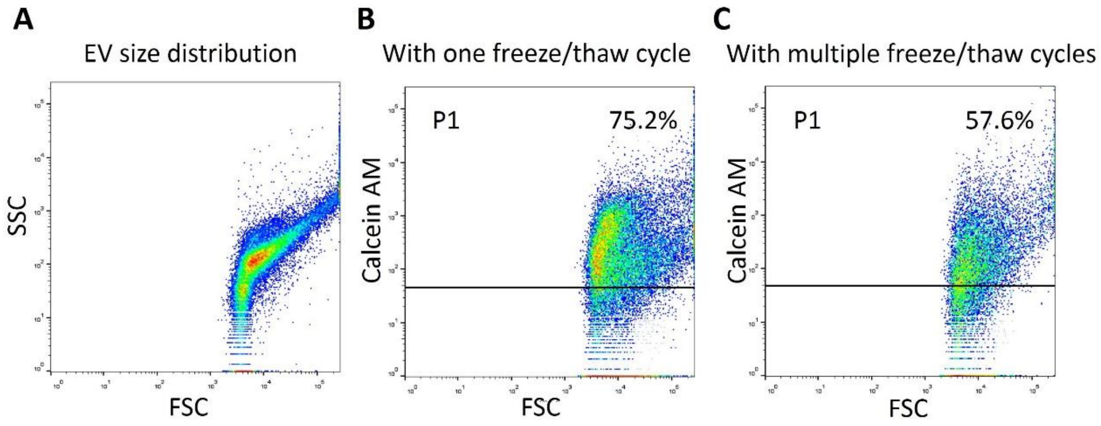

Fig. S2. Flow cytometric analysis of MSC-derived EVs. (A) Size distribution of EVs. (B) Percentage of calcein-AM-positive EVs after one freeze/thaw cycle. (C) Percentage of calcein-AM-positive EVs after multiple freeze/thaw cycles.

Table S1. Primers used in qRT PCR analysis.

Gene		Primer sequence (5'to 3')	Product
RUNX2	Forward	CCACAGAGCTATTAAAGTGACAGTG	87
	Reverse	AACAAACTAGGTTTAGAGTCATCAAGC	
OCN	Forward	AGCAGGAGGGCAGTAAGGTGGTGAA	196
	Reverse	ATGCCCTAAACGGTGGTGCCATAGA	
OPN	Forward	CTCAGAGGAGAAGGCGCATTG	221
	Reverse	TCTCTGCATGGTCTCCGTCGT	
GAPDH	Forward	GTCTTCACCACCATGGAGAAGG	197
	Reverse	TCATGGATGACCTTGGCCAG	
